# Utilize *in-vivo* offline PET/CT imaging to evaluate range deviations of implanted metal-clips in whole-breast proton radiotherapy

**DOI:** 10.1093/jrr/rraf041

**Published:** 2025-09-30

**Authors:** Fuquan Zhang, Yongkuan Hao, Yan Lu, Jiayi Guo, Rong Zhou, Yinxiangzi Sheng, Jiangang Zhang, Qing Zhang, Jingfang Zhao, Jingyi Cheng

**Affiliations:** Department of Nuclear Medicine, Shanghai Proton and Heavy Ion Center, Fudan University Cancer Hospital, 333 Huiyuan Road, Pudong District, Shanghai 201321, China; Shanghai Key Laboratory of Radiation Oncology, 333 Huiyuan Road, Pudong District, Shanghai 201321, China; Shanghai Engineering Research Center of Proton and Heavy Ion Radiation Therapy, 333 Huiyuan Road, Pudong District, Shanghai 201321, China; College of Physics, Sichuan University, No. 24 South Section 1, Yihuan Road, Wuhou District, Chengdu 610065, China; Key Laboratory of Radiation Physics and Technology, Ministry of Education (Institute of Nuclear Science and Technology), Sichuan University, No. 24 South Section 1, Yihuan Road, Wuhou District, Chengdu 610065, China; Department of Radiotherapy, Zhongshan Hospital, Fudan University, 180 Fenglin Road, Xuhui District, Shanghai 201321, China; Shanghai Key Laboratory of Radiation Oncology, 333 Huiyuan Road, Pudong District, Shanghai 201321, China; Shanghai Engineering Research Center of Proton and Heavy Ion Radiation Therapy, 333 Huiyuan Road, Pudong District, Shanghai 201321, China; Department of Radiotherapy, Shanghai Proton and Heavy Ion Center, 333 Huiyuan Road, Pudong District, Shanghai 201321, China; Shanghai Key Laboratory of Radiation Oncology, 333 Huiyuan Road, Pudong District, Shanghai 201321, China; Shanghai Engineering Research Center of Proton and Heavy Ion Radiation Therapy, 333 Huiyuan Road, Pudong District, Shanghai 201321, China; Department of Medical Physics, Shanghai Proton and Heavy Ion Center, 333 Huiyuan Road, Pudong District, Shanghai 201321, China; Department of Nuclear Medicine, Shanghai Proton and Heavy Ion Center, Fudan University Cancer Hospital, 333 Huiyuan Road, Pudong District, Shanghai 201321, China; College of Physics, Sichuan University, No. 24 South Section 1, Yihuan Road, Wuhou District, Chengdu 610065, China; Key Laboratory of Radiation Physics and Technology, Ministry of Education (Institute of Nuclear Science and Technology), Sichuan University, No. 24 South Section 1, Yihuan Road, Wuhou District, Chengdu 610065, China; College of Physics, Sichuan University, No. 24 South Section 1, Yihuan Road, Wuhou District, Chengdu 610065, China; Key Laboratory of Radiation Physics and Technology, Ministry of Education (Institute of Nuclear Science and Technology), Sichuan University, No. 24 South Section 1, Yihuan Road, Wuhou District, Chengdu 610065, China; Shanghai Key Laboratory of Radiation Oncology, 333 Huiyuan Road, Pudong District, Shanghai 201321, China; Shanghai Engineering Research Center of Proton and Heavy Ion Radiation Therapy, 333 Huiyuan Road, Pudong District, Shanghai 201321, China; Department of Medical Physics, Shanghai Proton and Heavy Ion Center, 333 Huiyuan Road, Pudong District, Shanghai 201321, China; Department of Nuclear Medicine, Shanghai Proton and Heavy Ion Center, Fudan University Cancer Hospital, 333 Huiyuan Road, Pudong District, Shanghai 201321, China; Shanghai Key Laboratory of Radiation Oncology, 333 Huiyuan Road, Pudong District, Shanghai 201321, China; Shanghai Engineering Research Center of Proton and Heavy Ion Radiation Therapy, 333 Huiyuan Road, Pudong District, Shanghai 201321, China; Shanghai Key Laboratory of Radiation Oncology, 333 Huiyuan Road, Pudong District, Shanghai 201321, China; Shanghai Engineering Research Center of Proton and Heavy Ion Radiation Therapy, 333 Huiyuan Road, Pudong District, Shanghai 201321, China; Department of Radiotherapy, Shanghai Proton and Heavy Ion Center, 333 Huiyuan Road, Pudong District, Shanghai 201321, China; Shanghai Key Laboratory of Radiation Oncology, 333 Huiyuan Road, Pudong District, Shanghai 201321, China; Shanghai Engineering Research Center of Proton and Heavy Ion Radiation Therapy, 333 Huiyuan Road, Pudong District, Shanghai 201321, China; Department of Medical Physics, Shanghai Proton and Heavy Ion Center, 333 Huiyuan Road, Pudong District, Shanghai 201321, China; Department of Nuclear Medicine, Shanghai Proton and Heavy Ion Center, Fudan University Cancer Hospital, 333 Huiyuan Road, Pudong District, Shanghai 201321, China; Shanghai Key Laboratory of Radiation Oncology, 333 Huiyuan Road, Pudong District, Shanghai 201321, China; Shanghai Engineering Research Center of Proton and Heavy Ion Radiation Therapy, 333 Huiyuan Road, Pudong District, Shanghai 201321, China

**Keywords:** PET verification, proton radiotherapy, offline PET, surgical clips

## Abstract

This study presented a quantitative analysis of the differences in the depths of the distal 50% of acquired and estimated positron emission tomography (PET) images for 18 patients who had a total of 109 titanium (Ti) metal-surgical clips implanted after breast-conserving surgery. Offline PET/computed tomography (PET/CT) images were acquired after proton irradiation. Hounsfield Unit modifications were applied to correct for metal artifacts induced by the Ti clips in the planning CT scans of the soft tissues surrounding the clips. The positron-emitting-isotope PET distribution was calculated through Range-Verification scripting. Quantitative analysis was conducted on the depth differences at the distal 50% R_50_ of the PET and the calculated PET distribution. Using the R_50_ method, the depth verification results of the clips and the normal tissues were compared. The R_50_ method calculates the positional difference at the half-maximum value 2 cm from the skin, with clips beyond this position not affecting the results. Analyses of the regions around the Ti clips were conducted. The depth difference for Ti < 2 cm (where the depth of the clips from the skin was <2 cm) was −1.63 ± 1.08 mm, while the corresponding normal tissue (Ti_cont_) showed a depth difference of −1.79 ± 1.15 mm. There was no statistically significant difference in the depth differences between Ti < 2 cm and the corresponding Ti_cont_. This study utilized offline PET verification to demonstrate that applying tissue corrections based on surgical clips and surrounding muscle tissues in clinical practice ensures that the presence of surgical clips does not compromise the precision of proton dose delivery at the surgical site.

## INTRODUCTION

Breast-conserving surgery combined with radiotherapy (RT) was recognized as an effective treatment option for breast cancer patients, significantly improving disease-free survival and overall survival rates [1]. To accurately localize targets in subsequent RT sessions, 3–5 titanium (Ti) clips were placed in the tumor bed during surgery. These clips served as landmarks to address the challenge posed by the non-rigidity of breast tissue and its potential movement and deformation relative to skeletal structures [2]. Utilizing these clips as reference points in RT planning demonstrated higher precision compared to relying solely on skeletal anatomy or breast surface markers [3, 4], resulting in more accurate irradiation and reduced local tumor recurrence rates [5–7].

However, the use of surgical clips presents challenges for accurate RT implementation, particularly in proton therapy. While proton therapy offers advantages such as sparing doses to cardiopulmonary structures and has seen increasing clinical adoption, it remains sensitive to respiratory motion and artifacts caused by high-density materials. Titanium, commonly used for surgical clips, induces significant radiographic artifacts on computed tomography (CT) images, which are critical for dose calculation in both proton and conventional radiation therapies. These artifacts may compromise the geometric accuracy of target delineation and range prediction, thereby affecting treatment plan integrity. The calculation of irradiation distance is constrained by inherent limitations in CT image acquisition, such as image noise and beam hardening issues, which can directly affect the depth of radiation exposure. Additionally, local volume effects and reconstruction artifacts introduce further complications, particularly with the presence of metal implants, which can produce significant reconstruction artifacts. The development of proton therapy plans requires converting CT values to proton stopping powers to calculate the dose deposition of the proton beam. Although algorithms for such calibration curves exist, the uncertainty in these curves is inevitable, especially since the conversion formula for protons depends on the atomic number of the material rather than electron density, and thus two materials with the same Hounsfield Unit (HU) value can have completely different proton stopping powers. Moreover, studies have shown [8] that the uncertainty in proton range estimation calculated using high-quality CT images and more accurate conversion formulas is ~3%. In proton therapy, this uncertainty in range can be addressed in various ways, such as using tissue correction methods. In clinical practice, tissue density correction is commonly applied to clips and the regions where they cause artifacts. Nevertheless, regardless of the approach, validation of these methods in patients through *in vivo* proton range verification is necessary.

*In-vivo* range monitoring is the most ideal way to verify beam range. One of the commonly used monitoring techniques is positron emission tomography (PET) [9]. Among all PET data acquisition methods, offline data acquisition is generally considered the most clinically applicable because it does not occupy treatment time and is cost-effective [10].

This study used offline PET methods [10–12] to analyze clinical cases. Additionally, a depth verification method was utilized to assess the impact of surgical clips on the range of the proton beam.

## MATERIALS AND METHODS

### Patient set and PET prediction

This retrospective study was approved by the Ethics Committee of Shanghai Proton and Heavy Ion Hospital, and the requirement for informed consent was waived. The study recruited 18 breast cancer patients who had undergone breast-conserving surgery and received adjuvant proton therapy at the Department of Proton and Heavy Ion Radiation Therapy, Shanghai Proton and Heavy Ion Hospital. All patients had signed informed consent forms before undergoing any examinations. Metal Ti clips were implanted in all 18 patients, resulting in a total of 59 clips placed, which produced 109 projections intersecting the Ti clips in various fields. PET validation scans were conducted after the initial proton radiation treatment in accordance with clinical diagnosis and treatment guidelines.

### Radiation therapy technique

All patients were immobilized in the supine position with their arms raised. RT plans were generated using the Syngo treatment planning system (VB13, Siemens Health Solution, Erlangen, Germany) employing a pencil beam algorithm. The plans utilized two non-coplanar beams, separated by 10° to 20°, with a fixed 45° beam nozzle. The prescription dose to the clinical target volume (CTV) was set at 44.055 Gy (relative biological effectiveness [RBE]-weighted) delivered over 15 fractions, while the simultaneous integrated boost (SIB) to the tumor bed (CTVboost) was 52.8 Gy (RBE-weighted). The CTV was defined to encompass the clinical breast region, with the anterior border as the skin, the posterior border excluding the chest muscle, chest wall muscle, and ribs, the lateral border as the axillary midline, and the medial border as the sternum and rib angle. The CTVboost was delineated to extend 1 cm beyond the tumor bed marked by clips. To account for lateral setup uncertainties, the planned target volume (PTV) was expanded radially by 5 mm beyond the CTV. To compensate for range uncertainties in the proximal and distal directions, Moyer's formula [13] was applied, following current clinical practice. Additionally, the PTV was carefully defined to be at least 1 mm away from lung tissue and to exclude the skin or ribs, aiming to minimize severe toxicity. It was ensured that the PTV and CTV received at least 95% and 99% of the prescription dose, respectively. The implanted clips were contoured with a HU value >1500 to represent Ti, while surrounding tissues were reclassified as adipose materials for tissue correction in the planning system.

Patient PET data were acquired using 'offline' PET, necessitating a transit time of ~5–10 minutes from the treatment room to the PET suite. The interval between treatment and PET scanning is incorporated as an input parameter in our analysis. This time interval significantly impacts the decay dynamics of various radioisotopes. Due to the unique decay constants of different isotopes, the length of this interval directly influences the activity of the radioisotopes, thereby affecting the accuracy of the predicted PET images. Our PET-RV algorithm has been designed to account for these variations, allowing for an accurate simulation of the actual radioactive distribution.

### PET prediction

Given the patient's CT data and treatment plan, the predicted PET image can be calculated by an analytical method [14]. For our study, we employed the PET-range verification (RV) program developed by Raysearch Laboratory in Sweden. As the dose distribution image from the treatment plan cannot be directly compared to the proton-induced radioactivity distribution image, the PET-RV program was used to generate a predicted proton-induced radioactivity distribution image according to the treatment plan. The RV script utilizes image information and plan details from RayStation to calculate the activity distribution information. The calculation method employed is numerical computation. Subsequently, this predicted image was compared to the proton-induced radioactivity distribution image obtained from the patient to determine the difference, regarded as the error value of the proton beam depth.

The PET-RV module employs a precomputed lookup table of PET data to replace conventional pencil-beam kernels in dose calculation. By rapidly retrieving activity distribution data based on proton energy and incident direction parameters, it reconstructs three-dimensional activity distribution through discrete scanning spot contribution superposition. This approach demonstrates superior computational efficiency compared to Monte Carlo simulations (<30 seconds per case vs hours-level computation), with accuracy primarily dependent on the precision of the precomputed data tables. Although Monte Carlo methods can concurrently obtain dose and isotope production distributions (e.g. O-15 and C-11 yields), their clinical implementation faces limitations due to dependency on nuclear reaction cross-section database completeness and prohibitive computational resource requirements for real-time verification.

Key parameter processing involves two critical aspects: (i) A temporal calibration module dynamically corrects activity decay across energy layers by analysing scanning spot delivery sequences, particularly accounting for extended decay time in earlier-delivered low-energy layers. (ii) The biological washout model remained disabled in current homogeneous polymethyl methacrylate (PMMA) phantom experiments (density 1.19 g/cm^3^, relative stopping power 1.156). Future *in vivo* applications will require organ-specific washout parameter mapping through HU-based tissue differentiation, addressing half-life variations between soft tissues and bone structures. This technical framework has been experimentally validated using phantoms at clinical facilities including Heidelberg Ion Beam Therapy Center, confirming its applicability for offline verification in proton therapy scenarios.

### PET acquisition and data preprocess

The proton beam was generated by a synchrotron linear accelerator (Siemens, Germany) with energy ranging from 48 to 221 MeV. PET/CT imaging was performed using Biograph mCT (Siemens, Germany) with four detection rings. Each ring contains 192/4 = 48 blocks, and each block contains 13 × 13 lutetium-oxyorthosilicate crystals with a crystal size of 4 × 4 × 20 mm. The X-ray tube voltages for CT were 80, 100, 120, and 140 kV. The reconstructed image had a pixel size of 4 mm × 4 mm and a thickness of 0.6 mm. The PET detection system had four detection rings, and the detector aperture was 78 cm with an axial field of view of 21.8 cm. The coincidence time window of the scanner was 4.1 ns, and the energy window was 435–650 keV. The TrueX algorithm was used for image reconstruction. Immediately after proton therapy, the patient was transported to the PET room with an average interval of 10.4 ± 3.3 minutes. Local PET/CT imaging was acquired for 30 minutes using a carbon fiber CT bed. The reconstructed PET image was based on the ^22^Na nuclear decay model to ensure full radionuclide count acquisition without time correction [15]. CT attenuation correction was applied during image reconstruction.

Automatic rigid registration was performed using the CT from the treatment plan and the CT from PET/CT, followed by manual adjustment to ensure complete registration of bony landmarks (ribs) in the radiation area.

The PET images were subjected to smoothing and normalization processes. Smoothing was performed using a sliding window method, employing specific parameters: window sizes of 3 × 3 or 5 × 5, a stride of 1, and boundary handling via zero padding. Normalization involved adjusting the scale of the measured PET image to match the activity level of the predicted image, while disregarding the numerical magnitude information. The normalization factor was determined by comparing the ratio of average activity in the target area between the measured and predicted PET images. Subsequently, the measured PET image was multiplied by this normalization factor to align the activity levels.

### Depth error verification

**Fig. 1:**
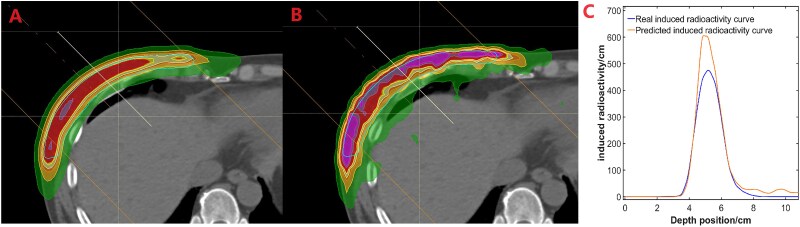
The comparison between the predicted and measured PET images. The profile was obtained from the localization CT image. The irradiation was conducted from the upper left corner towards the lower right direction, with the thick solid line in the image representing the field angles. Panel A shows the predicted distribution of positron emission, while panel B presents the measured distribution. Panel C exhibits the positron emission curves obtained by sampling along the thick white line from the predicted and measured images, which are used for calculating ΔR50. As can be observed in the figure, there are differences between the predicted and measured images in some regions. The emission values at the same spatial locations can be compared using the sampling lines, and the errors can be calculated accordingly.

The Planning Target Volume (PTV) and metal clips in the treatment plan were projected onto the two-dimensional plane perpendicular to the beam direction—termed the Beam Eye's View (BEV) [16]. These projections were designated as the PTV region and the Clips region, respectively. The sampling line, a straight line parallel to the beam direction crossing the PTV region [17], recorded the radioactivity at each spatial point along its path. By comparing the differences between the predicted and measured PET images at identical spatial positions along the sampling line, the depth error of the beam was determined. Given that the voxel size of the original PET data was 3 mm, the distribution interval of all sampling lines was set at 3 mm. The extent of the BEV was defined from −12 to 12 cm in both the x and y directions, with a spacing of 3 mm between sampling lines, resulting in a total of 6400 sampling points. These lines were evenly projected onto the BEV, and each line acquired a pair of data along the beam direction in both the predicted and measured PET images, constructing a spatial depth curve for calculating ΔR_50_, as depicted in Fig. 1.

Each sampling line generated a pair of spatial depth curves, as illustrated in Fig. 1. The definition of ΔR_50_ is as follows: (i) Record the maximum values of a pair of sampling lines passing through the same spatial point, calculate half of their maximal values V_50calc_ and V_50meas_, and denote the corresponding abscissas as R_50calc_ and R_50meas_ for V_50calc_ and V_50meas_, respectively. (ii) Calculate ΔR_50_ = R_50calc_ -R_50meas_ to obtain the depth difference of a pair of curves [18]. (iv) Within the BEV, each sampling line can obtain a ΔR_50_ value, and the mean and standard deviation of all ΔR_50_ values can be calculated to obtain the depth error value of the patient's proton therapy beam.

### Statistical analysis of clips region

**Fig. 2:**
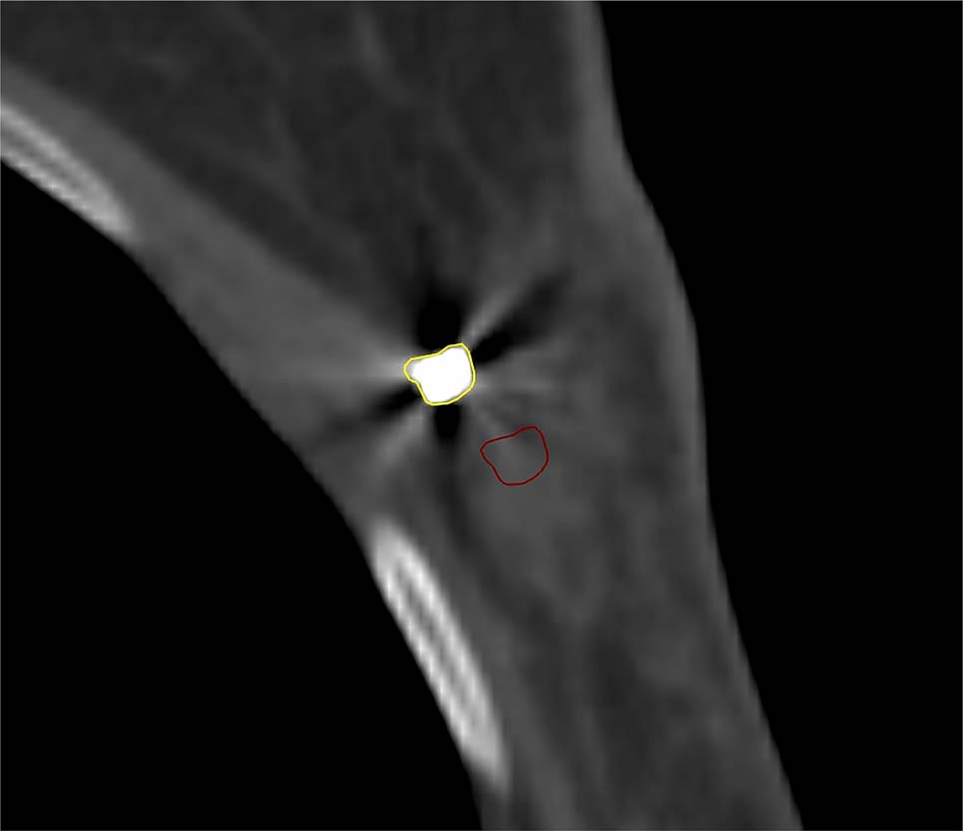
Comparison of depth differences between clips and normal tissue in proton therapy. The ROI in the upper left corner represents the clips and the normal tissue for comparison, while the ROI in the bottom right corner is the density correction area for normal tissue with metal artifacts.

The R50 method was used to calculate the positional difference at half of the maximum value. The location of half of the maximum value was observed 2 cm away from the skin along the beam direction. When clips were positioned beyond this half-maximum value on the sampling line, they did not influence the results of the R50 method. Thus, a boundary of 2 cm was established, and clips were categorized into two groups: those within a distance <2 cm from the skin, containing 44 clips, named Ti <2 cm, and those beyond 2 cm, containing 65 clips, named Ti >2 cm. Simultaneously, the depth differences between clips and normal tissue were compared. To ensure an equivalent area size for normal tissue as for clips, the ROI of clips was shifted 1 cm towards the center of the boost, named Ti_cont_, as shown in Fig. 2. The tissue composition at the Ti_cont_ displacement point was consistent with the surrounding tissue of the Ti clip, which was soft tissue. In this study, the initial interval between sampling lines was set at 3 mm. To enhance the resolution and accuracy of depth difference analysis within the clips, these sampling lines were interpolated to a finer 1 mm spacing. The average depth difference for each sampling line within the clips range was then calculated.

An independent Samples T-Test was performed on the obtained data to observe their correlation. When *P* < 0.05, it was considered that there was a significant difference between the two groups.

### Calculation of range difference in clips region

**Fig. 3:**
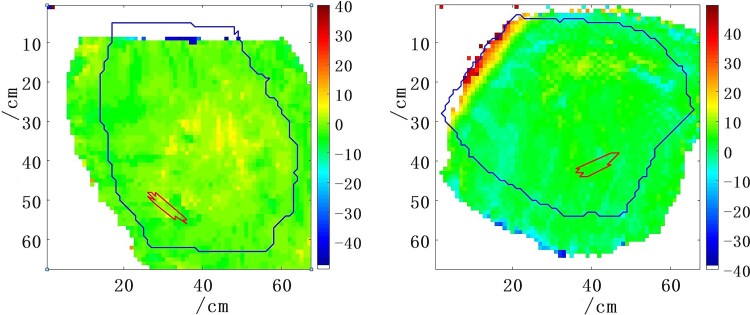
The distribution of beam range differences obtained on the BEV field of view. The two images correspond to the dual BEV fields of the same patient, with the red line indicating the projection of the Ti region on the BEV.

The distribution of range error corresponding to each BEV can be obtained based on the R_50_ method. When the clips region is projected onto the BEV, the projection range can be obtained. The range error within the projection range can be calculated to obtain the range error of this ROI, as shown in Fig. 3.

## RESULT

**Fig. 4:**
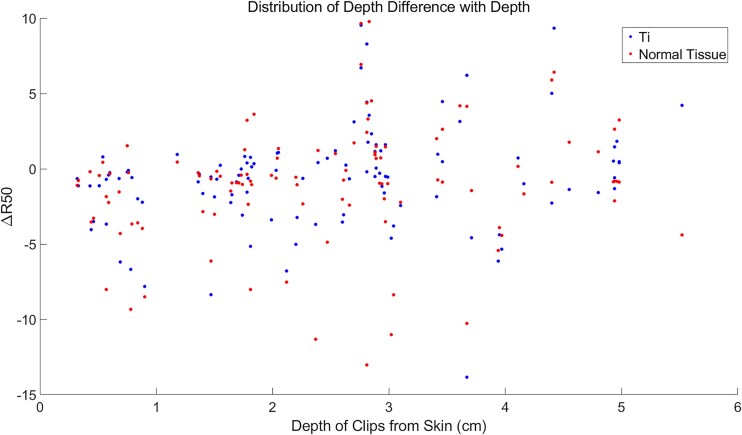
The average values of R50 for each clip, with the horizontal axis representing the clip's depth from the skin. There was no statistically significant difference in the depth between Ti <2 cm and Ti >2 cm.

The analysis of the region around the titanium (Ti) clips was conducted, as detailed in Table 1. The depth difference for Ti clips located <2 cm from the skin was −1.63 ± 1.08 mm, while the corresponding normal tissue (Ti_cont_) exhibited a depth difference of −1.79 ± 1.15 mm. For Ti clips situated >2 cm from the skin, the depth difference was 0.20 ± 2.10 mm, with the corresponding Ti_cont_ showing −0.99 ± 1.61 mm. There was no statistically significant difference in the depth differences between clips located <2 cm and those >2 cm from the skin, as depicted in Table 1 and Fig. 4.

**Table 1:** The comparison of depth difference between Ti_<2cm_ and Ti_>2cm_

	Ti_<2cm_(*n* = 44)	Ti_>2cm_(*n* = 65)	*P* value
Clips	−1.63 ± 1.08 mm	0.2 ± 2.1 mm	0.114
Ti_cont_	−1.79 ± 1.15 mm	−0.99 ± 1.61 mm	0.077
*P* value	0.767	0.511	

## DISCUSSION

As illustrated in Fig. 4, it is evident that not all mean values are distributed between −5 and 5 mm, primarily attributable to registration issues. A significant challenge with offline PET is achieving reproducible positioning during the PET scan. If the reproducibility of positioning is low, the accuracy of offline PET verification results is compromised. An effective solution involves having medical staff transport auxiliary positioning equipment from the proton therapy room to the PET room prior to the PET scan. However, in practice, the auxiliary positioning boards of some patients exceed the aperture of the PET/CT scanner. This discrepancy can worsen the registration between the planning CT and the PET/CT, leading to outliers in the R50 results. The analysis results of the metal Ti clips presented in this study confirm that the presence of Ti clips in the surrounding tissue does not negatively impact the actual proton therapy outcome in breast cancer treatment. In practice, smoothed offline PET images might not accurately reflect the activity levels within clipped regions. However, our study does not aim to directly evaluate the impact of clips on activity levels. We indirectly assess their potential influence by analyzing the positional accuracy of distal cutoff points. This indirect approach enables more precise evaluation of titanium clips' impact on proton therapy accuracy within the limitations of current technologies.

We proposed a method for classifying titanium (Ti) clips based on a 2 cm boundary from the skin, determined by the activity distribution in offline PET of the patient's body. In the offline PET method, the activity distribution and dose distribution are not entirely identical. Specifically, the R_50_ value of activity appeared 2–3 cm below the skin, and clips located beyond half of the maximum value did not affect the results of the R_50_ method. Therefore, we established 2 cm as the minimum boundary for classifying clips.

Parodi *et al.* [19] previously evaluated the impact of metal implants on the proton beam range in phantom model experiments and found that the deviation was within 1 mm with corrections applied. In this study, clinical case analyses were conducted based on Knopf's model experiments, and the results verified in patients were consistent with those from the model experiments. The depth verification results of Ti and surrounding tissues demonstrated that there was no significant difference in the distal attenuation position of the activity depth curve between the measured PET image and that calculated by the RV script in the presence of CT artifacts. This indicates that despite the proton beam passing through the location of clips, the delineation of clips and corrections made for the metal artifacts generated by clips within the normal tissue during treatment planning effectively reduced the uncertainty caused by CT artifacts. The findings demonstrate the robustness of PET measurements in monitoring proton therapy ranges, suggesting minimal interference from physical artifacts. Furthermore, the study reveals that proton traversal through larger metallic implants induces measurable range variations, consequently altering radiation dose distributions. This observation underscores the necessity of clinical validation for our methodology, given the critical importance of dosimetric precision in therapeutic applications. Through systematic analysis of range perturbations caused by metallic implants, our investigation substantiates the clinical applicability and measurement accuracy of offline PET systems for proton therapy verification. Notably, the consistent results across implants of varying sizes and material compositions provide robust empirical support for our methodology. These findings collectively demonstrate the feasibility and efficacy of utilizing offline PET technology for depth verification in proton therapy, even in anatomies containing metallic prostheses.

Several leading proton therapy centers are conducting studies on depth verification of the radiation therapy range. For example, Knopf *et al.* [20] studied 23 patients undergoing proton therapy using the PET depth verification method, covering tumors in the head and neck, thoracic spine, lumbar spine, and prostate regions, demonstrating that the average depth error for these patients was within 3 mm. Parodi conducted PET depth verification on nine patients with head and neck tumors undergoing proton therapy, with results showing that the depth error for head and neck tumors ranged between 1 and 2 mm. The results of this study are generally consistent with those of international peers. Differences in R50 are attributed to varying effects of factors such as biological washout, motion, and the limitations of Monte Carlo-simulated activity patterns [19].

## CONCLUSION

This article analyzed clinical cases and used a depth verification method that found the depth difference of Ti clips located <2 cm from the skin was −1.63 ± 1.08 mm, while the Ti_cont_ was −1.79 ± 1.15 mm. This study employed offline PET verification to demonstrate that applying tissue corrections based on surgical clips and surrounding muscle tissues in clinical practice ensures that the presence of surgical clips does not affect the accurate deposition of proton doses at the surgical site.

## Data Availability

Due to the strict data management requirements of the hospital, relevant data and materials will be provided to researchers as necessary.
